# Benzyl isothiocyanate provokes senolysis by targeting AKT in senescent IPF fibroblasts and reverses persistent pulmonary fibrosis in aged mice

**DOI:** 10.3389/fphar.2025.1506518

**Published:** 2025-05-02

**Authors:** Rui Wang, Fan Yang, Ying Liu, Meiting Peng, Lu Yu, Qinhui Hou, Yuan Liu, Zhenshun Cheng

**Affiliations:** ^1^ Department of Respiratory and Critical Care Medicine, Zhongnan Hospital of Wuhan University, Wuhan, China; ^2^ Wuhan Research Center for Infectious Diseases and Cancer, Chinese Academy of Medical Sciences, Wuhan, China; ^3^ Hubei Engineering Center for Infectious Disease Prevention, Control and Treatment, Wuhan, China

**Keywords:** idiopathic pulmonary fibrosis, cellular senescence, senolysis, benzyl isothiocyanate, fibroblasts

## Abstract

**Introduction:**

Senescent cells (SCs) accumulate with age and play a causative role in age-related diseases, such as idiopathic pulmonary fibrosis (IPF). Clearance of SCs attenuates lung fibrogenesis and favors fibrosis resolution, suggesting that targeting of SCs is recognized as a promising therapeutic approach for IPF. Isothiocyanates (ITCs) are natural compounds with anticancer and anti-aging properties, but their role in IPF remains unclear. The aim of our study to investigate whether benzyl isothiocyanate (BITC), a type of ITCs, can act as a senolytic agent thereby attenuating pulmonary fibrosis in aged mice.

**Methods:**

Primary lung fibroblasts from IPF patients and controls were cultured and treated with various ITCs to identify potential senolytic agents. Senescence-associated β-galactosidase staining, Cell viability assays, Annexin V/PI double staining, Caspase 3 activity assay, Western blot analysis, and qPCR were performed to evaluate senescence markers, cell viability, and apoptosis-related proteins after BITC treatment of senescent IPF lung fibroblasts *in vitro*. HE staining, Masson staining, Hydroxyproline assay, and Western blot analysis were used to assess the pathological progress, collagen content of lung tissues, and fibrotic gene expression changes after BITC treatment in C57BL/6 aged mice.

**Results:**

Using senescent IPF fibroblasts, we screened and identified BITC as a potent senolytic drug. We show that BITC selectively induces apoptosis in senescent IPF fibroblasts by targeting AKT signal pathway. Intraperitoneal administration of BITC to an age-related lung fibrosis mouse model effectively depleted senescent lung fibroblasts and reversed persistent pulmonary fibrosis.

**Discussion:**

Our study reveals that BITC may be a promising therapeutic option for IPF and other age-related disease that progress with the accumulation of senescent fibroblasts.

## 1 Introduction

Idiopathic pulmonary fibrosis (IPF) is a progressive and fatal chronic lung disease with unknow cause. The prognosis is poor, however, treatment options for IPF are still limited. Although the two antifibrotic agents Pirfenidone and Nintedanib, have demonstrated significant retardation in lung-function worsening, the mortality rate of IPF have scarcely reduced (King et al., 2014; Richeldi et al., 2014). Thus, there is a pressing need to develop novel therapeutic strategies for patients fighting with the devastating fibrotic lung disease.

IPF is a disease of aging. Cellular senescence, a critical phenotype of cellular aging, originally defined as stable cell cycle arrest in response to different stresses, is abundant within lung fibroblasts of human IPF and experimental pulmonary fibrosis (Álvarez et al., 2017). Senescent fibroblasts are characterized by elevated senescence-associated β-galactosidase activity, highly expressed the cell cycle suppressing proteins, including p21 and p16. Unlike normal cells, senescent fibroblasts usually exhibit a lower sensitivity to apoptotic stimuli and are viable for long periods of time (Lucas et al., 2023). In parallel, they remain metabolically active, secrete proinflammatory cytokines, chemokines, and extracellular matrix proteases, which collectively termed the senescence-associated secretory phenotype (SASP), robustly contributes the progressive fibrosis. Consequently, selective killing of senescent cells and inhibition of SASP have proved to be effective to attenuate the progression of pulmonary fibrosis and other age-related diseases (Baar et al., 2017; Chang et al., 2016; Ogrodnik et al., 2021).

Isothiocyanates (ITCs) are natural compounds that are found in cruciferous vegetables such as broccoli, cabbage or watercress. Some isothiocyanates, such as allyl isothiocyanate, benzyl isothiocyanate (BITC), indole-3-carbinol and sulforaphane (SFN), have been shown to reduce the risk of cancer occurrence and inhibit the growth of various human cancers (Dinh et al., 2021; Zhang et al., 2022). The mechanism of the ITCs anticarcinogenic activity is thought to be associated with the production of detoxifying enzyme, induction of apoptosis and inhibition of metastasis and angiogenesis. The relationship between tumors and cellular senescence is well-documented. Additionally, ITCs have been shown to be protective against various age-related diseases, including neurodegeneration (Santín-Márquez et al., 2019), kidney injury (Monteiro et al., 2023), and liver fibrosis (Kim et al., 2018). Recent studies have been reported that SFN and extracts of brassica chinensis could enhance antioxidant activity and delayed aging in *Caenorhabditis elegans* (Chen et al., 2016; Qi et al., 2021). However, the role of ITCs in cellular senescence and age-related lung fibrosis remains poorly defined.

In this study, we tested several ITCs using senescent IPF fibroblasts and identified BITC as a senolytic agent. BITC selectively induced senescent IPF fibroblasts apoptosis by inhibiting Akt signal pathway. Moreover, we found that BITC can effectively reversing persistent lung fibrosis and eliminating senescent fibroblasts in aged mice. Taken together, these results suggested that BITC could be a novel therapeutic approach for the treatment of lung fibrosis.

## 2 Materials and methods

### 2.1 Human primary lung fibroblast cultures and procedures

Human lung tissue from three IPF patients and age-matched normal peripheral tissue from three patients with pulmonary bullae were obtained from Zhongnan Hospital of Wuhan University. Diagnosis of IPF was made by multidisciplinary team in the department of Respiratory and Critical Care Medicine according to standards accepted by ATS/ERS/JRS/ALAT. This study was officially approved by the Ethics Committee of the Zhongnan Hospital of Wuhan University.

Human lung fibroblasts from IPF patients and controls were isolated and culture as follows. After washing with PBS, the lung tissues were cut into small pieces and then attached to the wall of cell culture dishes containing 1.5 mL Dulbecco’s modified Eagle’s medium (DMEM) containing 10% fetal bovine serum (FBS, Gibco, cat#:16000044) at 37°C in 5% CO_2_. After approximately 2 weeks culture, the fibroblasts could be harvested. Growth curves were constructed by seeding in triplicates 3 × 10^5^ cells/well in 6-well plates and counting them after trypsinization every 24 h for 5 consecutive days. For determination of population doubling levels (PDLs), cells were counted when reached confluency and again seeded at a density of 3 × 10^5^ cells/well. This was continued over 100–150 days. PDL was calculated as PDL = 3.2 × (log_haversted cells_-log_seeded cells_)+actual passage number (Hartmann et al., 2023).

For ITCs screening, Normal and IPF human lung fibroblasts at passage 12 were stimulated with different ITCs, including SFN (MCE, cat#: HY-13755), iberin (MCE, cat#: HY-101413), 4-pentenyisothiocyanate (MCE, cat#: HY-W269799) and BITC (MCE, cat#: HY-77813). The isothiocyanates were dissolved in DMSO. FOXO4-DRI (NovoPro, cat#: 318716) was dissolved in PBS. After 48 h, the cells were collected and analysis.

### 2.2 Senescence-associated β-galactosidase staining

Cells were performed with a senescence-associated β-galactosidase (SA-β-Gal) staining kit (Beyotime, cat#: C0602) according to the manufacturer’s protocol. Cells were washed three times with PBS, fixed with β-galactosidase staining fixative for 15 min at room temperature and incubated with the staining working solution at 37°C overnight. Images were obtained using an Olympus IX73 microscope with 10 × objective (Olympus, Japan). Positive cell numbers were quantified using ImageJ software.

### 2.3 Cell viability assays

Cell viability of cells was detected using the cell counting Kit-8 (Biosharp, cat#: BS350A) following manufacturer’s instructions and absorbance was recorded at 450 nm by using a VICTOR Nivo Multiode Plate Reader (PerkinElmer, Finland).

### 2.4 Annexin V/PI double staining

The proportion of apoptotic cells was determined using Annexin V-FITC/PI Apoptosis Kit (Multisciences, cat#: AP101) and following the manufacturer’s instructions. Lung fibroblasts were washed twice with PBS and treated with trypsin. Following a 5 min centrifugation at 600 *g*, pelleted cells were resuspended in 1X Annexin-Binding Buffer and then incubated with 10 μL PI and 5 μL Annexin V-FITC for 10 min at room temperature in the dark. The apoptotic cells were detected using a Gallios Flow Cytometer (Beckman Coulter, United States).

### 2.5 Caspase 3 activity assay

Caspase 3 activity was detected using a Caspase 3 assay Kit (Beyotime, cat#: C1116). Briefly, cells were collected and centrifuged at 600 *g* for 5min and washed with PBS. The washed cells were resuspended in cell lysis buffer and immersed on ice for 15 min to obtain the cell lysates. The cell lysates were mixed with Ac-DEVD-pNA at 37°C for 2 h, and then read with a VICTOR Nivo Multiode Plate Reader (PerkinElmer, Finland) at a wavelength of 405 nm.

### 2.6 Western blot analysis

Western blot analysis of protein expression was performed as previously described. After blocking with 5% milk, the membranes were incubated with the primary antibodies as follows: β-actin (1:50,000, Abclonal, cat#: AC026), p21 (1: 2000, Proteintech, cat#: 10355-1-AP), p16 (1:1000, CST, cat#: 80772), BAX (1:1000, Abclonal, cat#: A19684), BCL2 (1:1000, Abclonal, cat#: A20777), Cleaved Caspase 3 (1:1000, CST, cat#: 9664), AKT (1:5000, Abclonal, cat#: A18675), pAKT (1:750, Abclonal, cat#: AP1208), α-SMA (1:1000, Abcam, cat#: ab124964), COL1A1 (1:2500, Abclonal, cat#: A16891), and COL3A1 (1:1000, Abclonal, cat#: A0817). Protein bands were visualized using an enhanced chemiluminescence system (Tanon, China) and band intensities from both methods were analyzed using ImageJ. All the original images were presented in the Supplementary Material.

### 2.7 Quantitative real-time RT-PCR (qPCR)

Total RNA was isolated with TRIzol (Invitrogen, United States) according to the manufacturer’s instructions, and cDNA was generated using a Fast King RT Kit with gDNase (TIANGEN Biotech, China). qPCR was performed using UltraSYBR (CWBIO, China) and human or mouse β-actin as an internal control. Primers are listed in Supplementary Table S1.

### 2.8 Animal experiments

Male C57BL/6 aged mice (18 months) and young mice (3 months) were purchased from Gempharmatech Animal Centre (Jiangsu, China). All the mice were housed in a specific pathogen-free (SDF) conditions, at 25°C ± 2°C and 55% ± 5% humidity on a 12 h light-dark cycle, and fed same diet. Mice were anesthetized with pentobarbital (50 mg/kg) and orotracheally intubate under the guidance of a laryngoscope. Bleomycin (BLM) (1.5 mg/kg; Hisun Pfizer) or saline was injected into the tracheal tube (Liu et al., 2023). BITC was prepared by dissolving it in a solution of 10% DMSO and 90% corn oil. In young mice, BITC (12.5 mg/kg) or vehicle was administered intraperitoneally once daily, starting 7 days after BLM treatment, and continued for 2 weeks. In aged mice, BITC (12.5 mg/kg) or vehicle was administered intraperitoneally once daily, starting 3 weeks after BLM treatment, and continued for 3 weeks. All animal experiments were performed after review by and approval from the Animal Studies Committee of the Zhongnan Hospital of Wuhan University.

### 2.9 Hydroxyproline assay

The hydroxyproline content was quantified using a Hydroxyproline assay Kit (Jiangcheng, cat#: A030-2) as previously described (Hu et al., 2022).

### 2.10 Histopathology and immunofluorescence

The lung samples were fixed with 4% formaldehyde, followed by dehydration and embedding in paraffin. The tissues were cut into 4-μm-thick transverse sections. Then, hematoxylin and eosin (HE) and Masson’s trichrome staining were performed using standard techniques. For immunofluorescence, studies used antibodies against p16 (Abcam, cat#: ab241543) and Vim (CST, cat#: 5741). Negative controls were performed by replacing the primary antibody with a nonspecific IgG of the same species. Immunofluorescence analysis was performed using six under 40 × magnification for one section per individual from each group. Positive cell numbers were acquired using ImageJ software.

### 2.11 Statistical analysis

Data were statistically analyzed using Prism 8.0 software (Graph Pad). Differences between two groups were calculated by two-tailed Student’s t*-*test. Comparisons of more than two groups were performed using one- or two-way ANOVA analysis. Results are presented as mean ± SEM, and *P* < 0.05 was considered significant.

## 3 Results

### 3.1 Identification of BITC as a senolytic agent in senescent IPF fibroblasts

**FIGURE 1:**
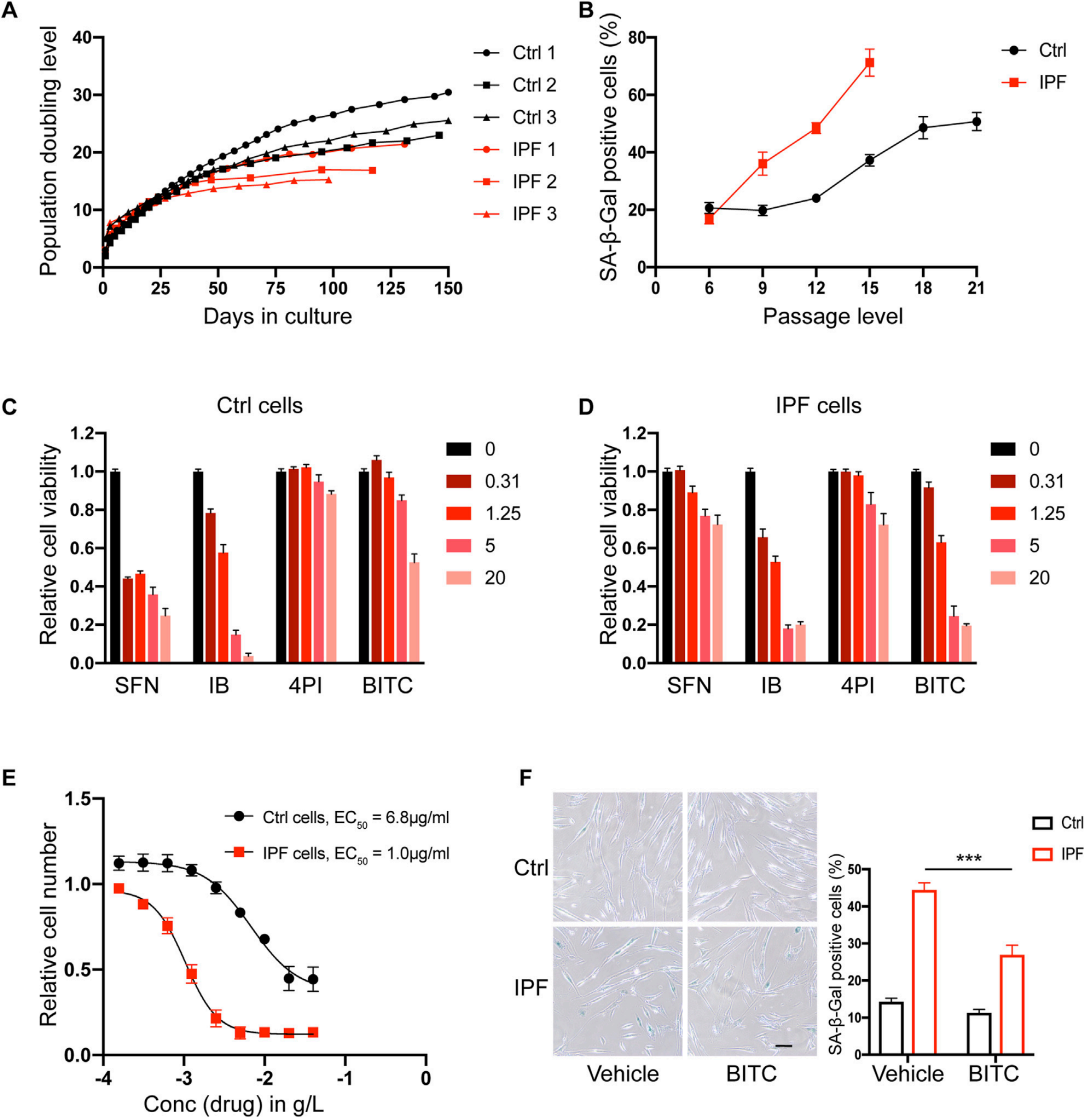
BITC selectively kill senescent IPF fibroblasts. **(A)** Replicative potential of primary human lung fibroblasts from control donors (Ctrl 1, Ctrl 2 and Ctrl 3) and IPF donors (IPF 1, IPF 2 and IPF 3). Cells were cultured under stable conditions and population doubling level (PDL) was observed for max 150 days. **(B)** Quantification of SA-β-Gal positive cells during the passage cultures. **(C, D)** Cell viability assays. Four ITCs were added to cultures of **(C)** non-senescent control and **(D)** senescent IPF primary lung fibroblasts at 4 concentration (0.31–20 μg/mL). **(E)** Dose response analysis of BITC. Increasing concentrations of BITC were tested and plotted against the remaining senescent IPF (red) and control cells (black) after 48 h treatment. **(F)** Representative images and quantification of SA-β-Gal positive cells from control and senescent IPF after BITC treatment 48 h. Scale bar, 20 μm. Error bars indicate mean ± SEM. Significant differences were assessed by two-way ANOVA. ***P < 0.001.

Previous study has found that IPF lung fibroblasts are characterized by an accelerated senescence phenotype (Hohmann et al., 2019). In the present study, primary lung fibroblasts derived from three control and three IPF patients (Supplementary Table S2) were cultured and passaged to induce senescence. Consistent with previous study, IPF cells in these cultures exhibited a slower growth rate and stopped proliferating earlier than control cells in terms of cumulative population doublings or passage levels (Figure 1A; Supplementary Figure S1). Furthermore, the fraction of senescence-associated β-galactosidase (SA-β-Gal) positive cells increased earlier in IPF cells, and determined that 50% of IPF cells were senescent at passage 12 (p12) (Figure 1B).

To examine the anti-senescence activity of ITCs, four common compounds with different potencies were selected, including SFN, iberin (IB), 4-pentenyisothiocyanate (4PI) and BITC. The chemical formulas of these compounds are shown in Supplementary Figure S2. Senescent IPF cells and non-senescent control cells at p12 were treated with four different concentration of each drug for 48 h and their viability were measured. Among them, only BITC was able to reduce the viability of senescent IPF fibroblasts specifically at a concentration of 1.25 μg/mL without significantly affecting the viability of health control cells (Figures 1C,D). Moreover, BITC showed a 6.8-fold lower EC50 values on senescent IPF cells compared to overall cell death (Figure 1E). To further validate this result, BITC were administrated at a concentration of 1.25 μg/mL and SA-β-Gal positive cells were calculated. We found that BITC can selectively and potently reduce the number of senescent IPF cells yet be safe to control cells (Figure 1F). Consequently, in the follow-up experiments, BITC was specifically applied to the fibroblasts derived from IPF.

### 3.2 Reduction of senescence markers by BITC in senescent IPF lung fibroblasts

**FIGURE 2:**
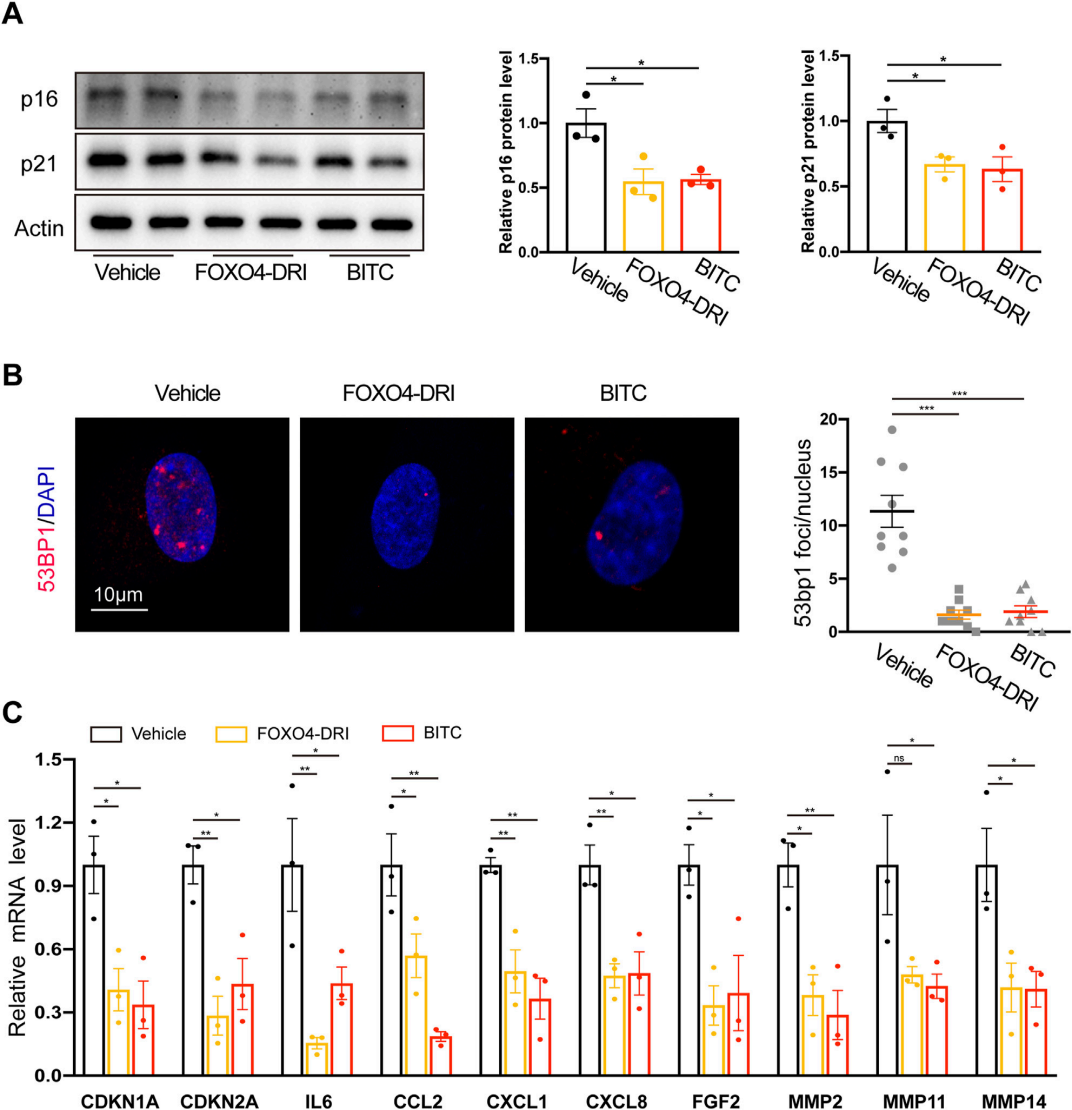
Multiple senescence markers are reduced in senescent IPF fibroblasts after treatment with BITC. Senescent IPF fibroblasts from three donors were added with FOXO4-DRI (25 μmol/L) and BITC (1.25 μg/mL) for 48 h and collected for multiple senescence analysis. **(A)** Representative images and quantification of p21 and p16 protein levels by Western blotting. **(B)** Representative images and quantification of the number of 53BP1 foci per cell. Scale bar, 10 μm. **(C)** qPCR analysis of the expression of CDKN1A, CDKN2A and eight common SASP genes, including IL6, CCL2, CXCL1,CXCL8, FGF2, MMP2, MMP11, and MMP14. Error bars indicate mean ± SEM. Significant differences were assessed by one-way ANOVA. *P < 0.05. ***P < 0.01. ***P < 0.001.

To further confirm and extend our results, the senolytic activities of BITC were compared to previously reported senolytic drugs FOXO4-DRI (a FOXO4 peptide which perturbs the FOXO4 interaction with p53) (Baar et al., 2017). BITC treatment in senescent IPF cells significantly decreased the expression of cell cycle regulators p16 and p21, and this inhibition was on par with the FOXO4-DRI (Figure 2A). In line with these findings, CDKN1A and CDKN2A mRNA expression was also reduced by treatment with BITC (Figure 2C). 53BP1 is recruited in response to DNA damage and accumulated in senescent cells (Oda et al., 2023). Compared to the health controls, significantly 53BP1 foci formation was observed in IPF fibroblasts and increased with passages (Supplementary Figure S3). Notably, 53bp1 foci formation was abolished upon BITC treatment or FOXO4-DRI treatment (Figure 2B), suggested that BITC provokes senolysis likely through blocking the recruitment of 53BP1 to DNA damage sites.

A key phenotypic characteristic of senescent cells is the acquisition of SASP, which contributes positively lung fibrosis development. We showed that core SASP genes such as canonical cytokines (IL6), chemokines (CCL2, CXCL1 and CXCL8), growth factors (FGF2), and matrix proteases (MMP 2, 11, and 14) were reduced in both two treatment (Figure 2C). Taken together, these results further confirmed the inhibition of senescence by BITC in senescent IPF fibroblasts.

### 3.3 BITC targets senescent IPF fibroblasts for Bax-dependent apoptosis

**FIGURE 3:**
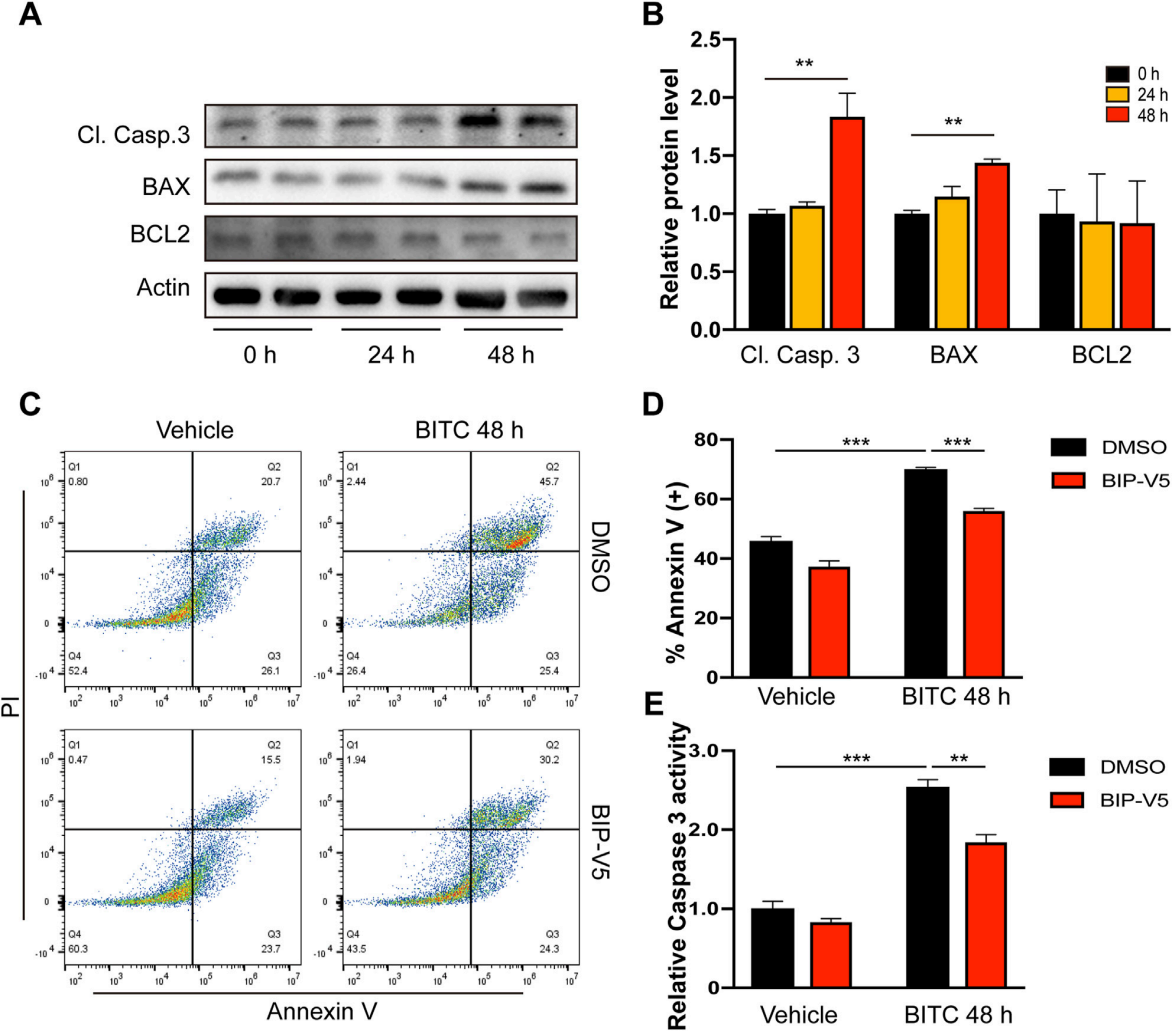
BITC targets senescent IPF fibroblasts for Bax-dependent apoptosis. **(A, B)** Senescent IPF fibroblasts were treated with BITC for 24 h and 48 h, the expression of cleaved caspase 3 (Cl. Casp.3), BAX and BCL2 protein levels were detected by Western blotting. **(C, E)** Cells were added with BIP-V5 (10 μM) and treated with BITC for 48 h, then **(C, D)** fixed with Annexin V/PI staining for the apoptosis rate analysis and (E) assessed with caspase 3 activity. Error bars indicate mean ± SEM. Significant differences were assessed by one-way ANOVA or two-way ANOVA. **P < 0.01. ***P < 0.001.

To further explored how BITC selectively kill senescent IPF lung fibroblasts, apoptotic-related molecules such as BCL2, BAX, and cleaved caspase 3 (Cl. Casp. 3) were examined by Western blot analysis. BITC treatment significantly induced a cleaved caspase 3, followed by subsequent upregulation of proapoptotic BAX without affecting anti-apoptotic BCL2 (Figures 3A,B). To determine whether BITC-induced apoptosis was BAX-dependent, the effects of exposure to BITC on cell apoptosis were evaluated after preincubation with the BAX-inhibiting peptide V5 (BIP-V5) (Huang et al., 2022). BITC treatment significantly increased fractions of apoptotic cells compared with vehicle, while addition of BIP-V5 resulted in increased cell survival as compared to BITC treatment alone (Figures 3C,D). Additionally, BITC induced lower levels of caspase 3 activity in BIP-V5-pretreatment cells than controls (Figure 3E). Together, these data indicated that BITC induces senescent IPF fibroblasts apoptosis in a BAX-dependent manner.

### 3.4 BITC provokes senolysis through attenuation of AKT signal pathway

**FIGURE 4:**
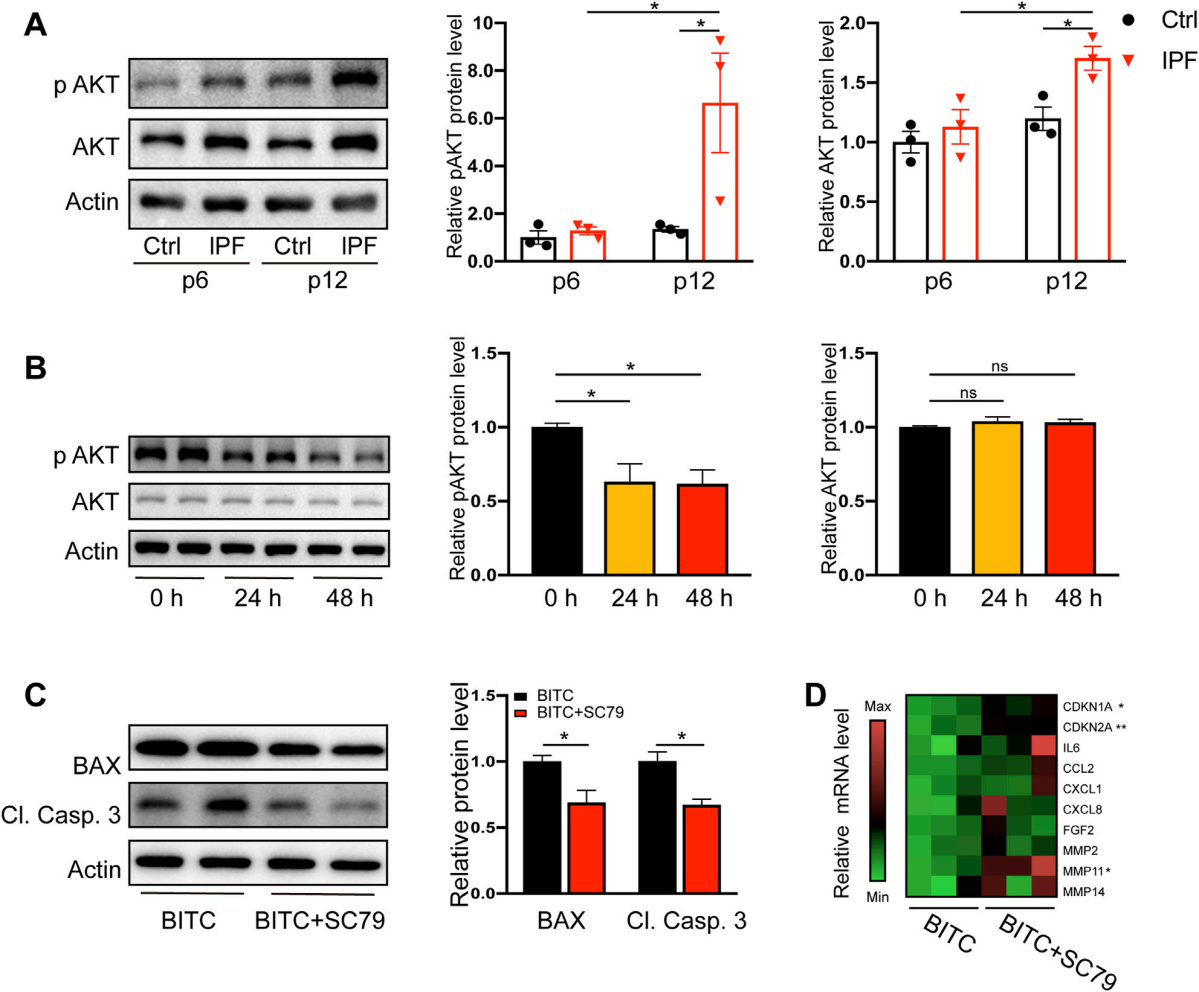
BITC provokes senolysis by attenuation of AKT signal pathway. **(A)** Representative images and quantification of AKT and phosphorylated AKT (p AKT) protein levels by Western blotting in human primary lung fibroblasts at early passage level (p 6) and late passage level (p 12). **(B)** Senescent IPF fibroblasts were treated with BITC for 24 h and 48 h, the expression of AKT and p AKT protein levels were detected by Western blotting. **(C)** Cells were added with SC79 (10 μM) and treated with BITC for 48 h, then the expression of BAX and cleaved caspase 3 (Cl. Casp. 3) protein levels were detected by Western blotting. **(D)** Gene expression heatmap for senescence and SASP genes in BITC-treated cells compared with BITC-treated cells added with SC79. Error bars indicate mean ± SEM. Significant differences were assessed by one-way ANOVA or two-way ANOVA. *P < 0.05. **P < 0.01. ns = no significant.

Numerous studies demonstrated that cell survival or anti-apoptosis can be mediated by an AKT-dependent pathway via impacting BAX and other targets (Kennedy et al., 1997; Yamaguchi and Wang, 2001). AKT and its activated form p-AKT (Ser473) were significantly higher in late passage IPF fibroblasts compared to controls (Figure 4A). BITC was sufficient to reduce the level of p-AKT in senescent fibroblasts by 24 h and the reduction lasted for at least 48 h (Figure 4B). In contrast, the expression of total AKT was not altered by BITC. These data suggested that AKT inactivation may account for induction of apoptosis in BITC treated cells. To further test it, cells were treatment with SC79 (Xu T. Y. et al., 2023), a known AKT signaling pathway inducer, to restore the level of p-AKT. SC79 treatment for 24 h sufficiently decrease the elevated expression of BAX and cleaved caspase 3 in BITC treated cells (Figure 4C). Furthermore, the expression of a series of cell cycle regulators and SASP genes was upregulated by the SC79 treatment in BITC treated cells (Figure 4D). These data suggested that AKT signal pathway plays a pivotal role in the senolytic mechanism of BITC.

### 3.5 BITC treatment promotes the resolution of established lung fibrosis in aged mice

Our next objective was to investigate the effects of BITC on lung fibrosis *in vivo*. We first studied its impact on experimental pulmonary fibrosis in young mice but found no significant effect of BITC on BLM-induced fibrosis (Supplementary Figure S4). Given that IPF is an age-related disease and previous studies have shown that fibrosis in aged mice following lung injury is persistent and associated with senescence and apoptosis resistance in lung fibroblasts (Hecker et al., 2014; Liu et al., 2023), we subsequently evaluated the efficacy of BITC in aged mice.

**FIGURE 5:**
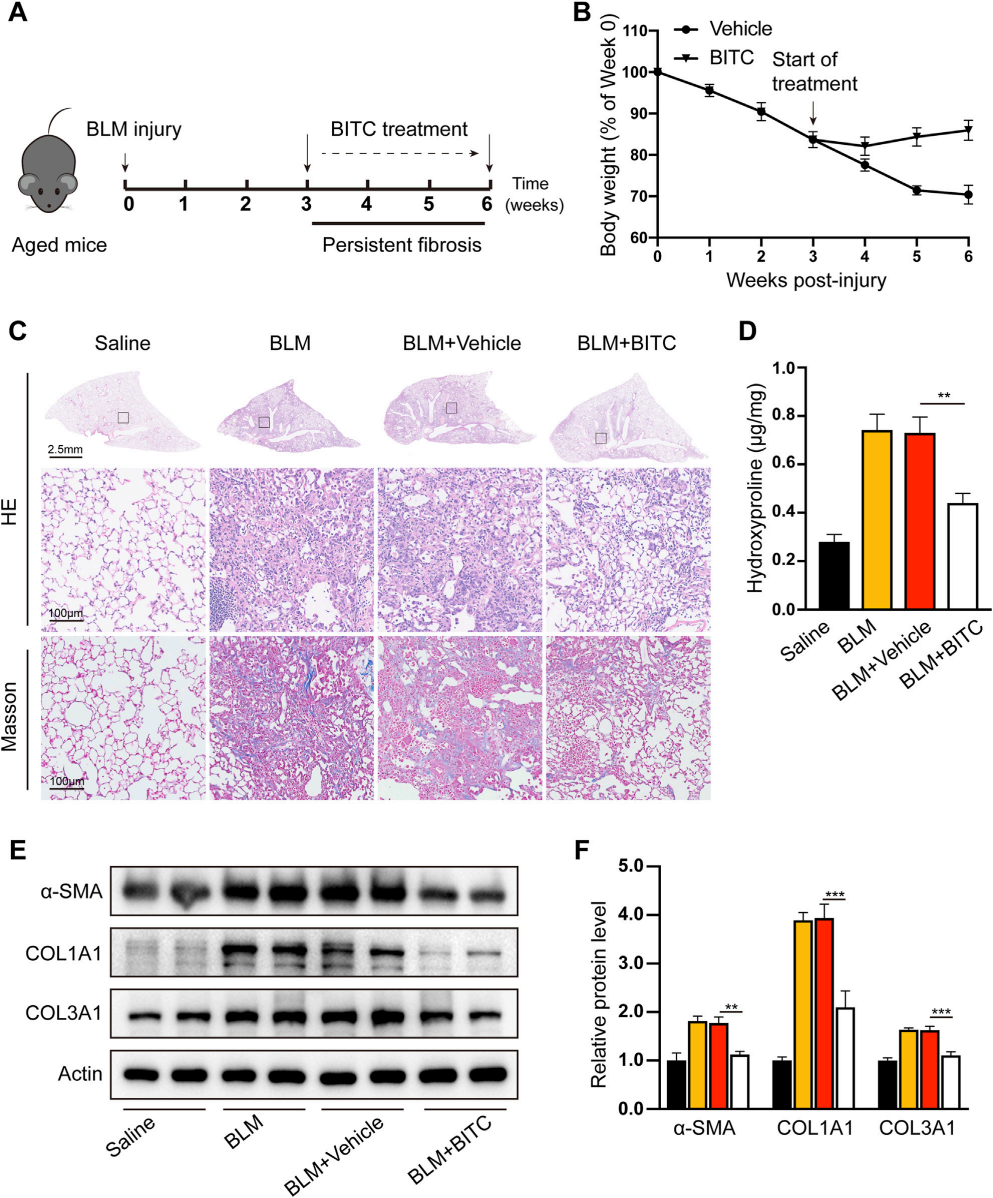
BITC reversed bleomycin-induced persistent pulmonary fibrosis in aged mice. **(A)** Modeling diagram. **(B)** Changes of body weight. **(C)** Representative images of hematoxylin and eosin (HE) and Masson staining. For HE staining: scale bar, 2.5 mm for top panel and 100 μm for bottom panel, which shows a higher magnification image of the boxed area in the top panel. For Masson staining: scale bar, 100 μm. **(D)** The hydroxyproline content in the lung tissues. **(E, F)** Representative images and quantification of collagen type I (COL1A1), collagen type III (COL3A1), and α-smooth muscle actin (α-SMA) levels by Western blotting. Error bars indicate mean ± SEM. n = 6 per group. Significant differences were assessed by two-way ANOVA. *P < 0.05. **P < 0.01.

Aged (18 months) mice were exposed single-dose BLM to establish persistent and irreversible lung fibrosis. Three weeks after BLM injury (when aged mice exhibit persistent fibrosis), mice were either treated with vehicle or treated with intraperitoneal injection of BITC once daily for three consecutive weeks. Six weeks later, mice were sacrificed and their fibrotic response were analyzed. The experimental timeline is shown in Figure 5A. Recovery to baseline weights was observed in BITC-treated mice, whereas vehicle-treated mice remained below baseline levels throughout the 6-week observation period (Figure 5B). H&E staining and Masson staining are commonly used to assess the severity of lung injury and the extent of fibrosis in lung tissues sections. As shown in Figure 5C, BITC alleviated BLM-induced lung tissue destruction and reduced collagen deposition compared with the vehicle group. Hydroxyproline assay is a highly recommended method for collagen content determination. We found that BITC significantly decreased hydroxyproline levels in lung tissue compared with the vehicle group (Figure 5D). These data suggested that BITC treatment caused alleviations that are both lung-specific and systemic in aged mice.

Myofibroblast persistence tightly parallels fibrosis in BLM-induced aged mice. We evaluated myofibroblast persistence after BITC treatment. Western blotting of mouse homogenates further revealed lower expression of α-SMA, COL1A1 as well as Col3A1 (Figures 5E,F). These data further suggest that BITC can resolve preexisting fibrosis or block the progression of fibrosis in aged mice.

### 3.6 BITC treatment eliminates senescent fibroblasts in BLM-induced aged mice

**FIGURE 6:**
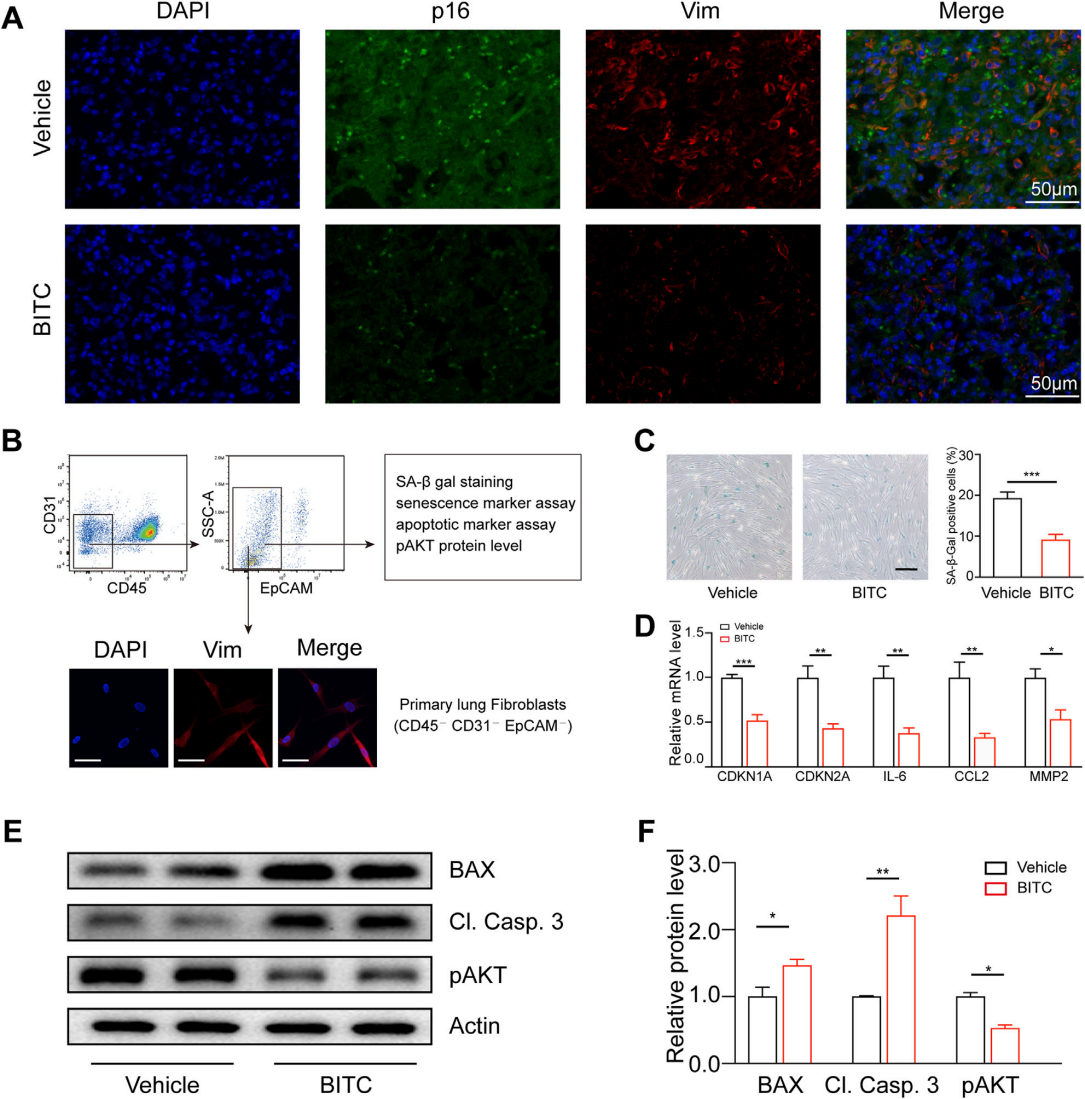
BITC treatment eliminates senescent fibroblasts in BLM-induced aged mice. **(A)** Representative images of p16 (green) and vimentin (Vim) (a biomarker for lung fibroblasts) (red) double-immunofluorescence staining of Vehicle-treated and BITC-treated aged fibrotic lungs. Scale bars, 50 μm. **(B)** Gating strategy for analysis and isolation of primary lung fibroblasts by flow cytometry (top) and representative images showing immunostaining for Vim. Scale bars, 20 μm. **(C)** Representative images and quantification of SA-β-Gal positive cells in sorted primary lung fibroblasts from Vehicle-treated and BITC-treated aged fibrotic mice. Scale bar, 20 μm. **(D)** qPCR analysis of the expression of CDKN1A, CDKN2A and three common SASP genes in sorted primary lung fibroblasts. **(E, F)** Representative images and quantification of BAX, cleaved caspase 3 (Cl. Casp. 3), AKT phosphorylated AKT (p AKT) protein levels by Western blotting in sorted primary lung fibroblasts. n = 6 per group for A, B, D; n = 3 per group for **(C, E, F)**. Error bars indicate mean ± SEM. Significant differences were assessed by unpaired two-tailed Student’s t*-*test. *P < 0.05. **P < 0.01. ***P < 0.001. ns = no significant.

To investigate whether the therapeutic efficacy of BITC in this model was due to the reduction of senescent cells in the lungs of mice, we measured the expression of senescence biomarkers in the lung fibroblasts. Immunofluorescence staining indicated that the numbers of p16 and vimentin (a marker of fibroblast) double-positive cells were markedly reduced by BITC treatment (Figure 6A). In addition, we studied the effects of BITC treatment on fibroblasts isolated from the lungs of BLM-induced aged mice (Figure 6B). BITC treatment resulted in a decreased number of SA-β-Gal positive cells in isolated lung fibroblasts cultures compared to those from Vehicle-treated aged mice (Figure 6C). Additionally, BITC treatment also reduced the levels of CDKN1A, CDKN2A and SASP factors in isolated lung fibroblasts (Figure 6D). This reduction was associated with a significant decrease in phosphorylated AKT and enhancements in cleaved caspase 3 and BAX expression (Figures 6E,F). These findings suggested that BITC treatment of BLM-induced aged mice clears senescent fibroblasts during fibrosis resolution.

## 4 Discussion

Fibroblast senescence is increasingly recognized as a feature of IPF. In mouse models of lung fibrosis and precision-cut lung slices from IPF patient *ex vivo*, selective removal of p16-expressing senescent fibroblasts or senolytic treatments attenuate fibrotic phenotype, suggesting that senescent fibroblasts participate in key aspects of lung fibrosis (Cooley et al., 2023; Lee et al., 2024). Given that primary lung fibroblasts derived from patients with IPF exhibited an accelerate phenotype, we utilized senescent IPF fibroblasts and passage-matched controls for testing several bioactive phytochemicals in cruciferous vegetables, including SFN, IB, 4PI and BITC, and finally identified BITC as a promising senolytic drug. We further investigated the mechanism and found that BITC exerts its senolytic effects by promoting BAX-dependent apoptosis and inhibiting AKT-related survival signaling pathways. Additionally, BITC promotes the resolution of lung fibrosis and exhibits senolytic activity on fibroblasts in bleomycin-induced aged mice.

BITC is one of the most studied naturally occurring ITCs present in cruciferous vegetable such as watercress, cabbage, cauliflower, kale and broccoli. BITC, like other ITCs, exhibits anti-bacterial, anti-fungal, anti-inflammatory and anticancer properties, as evidenced in preclinical studies (Dinh et al., 2021; Sundaram et al., 2022). In this study, although both BITC and IB exhibited properties of inducing death in senescent IPF fibroblasts, IB also showed cytotoxic effects on normal fibroblasts when inducing death in senescent IPF fibroblasts. In contrast, BITC preferentially killed senescent IPF fibroblasts, suggesting the importance of the aromatic ring in its senolytic activity. Although further study is needed to clarify the underlying mechanism, it is noteworthly that regulation of cellular senescence depends on the structure of the ITCs.

Senescent cells, like cancer cells, are known to employ anti-apoptotic and pro-survival pathway involved in maintaining the senescent state. The mechanism of a senolytic drug depends on inducing apoptosis in senescent cells either by inhibiting anti-apoptotic proteins or by stimulating the expression of pro-apoptotic molecules (Kirkland and Tchkonia, 2020). In the present study, we reveal that BITC induced apoptosis in senescent IPF fibroblasts by upregulating the expression of cleaved caspase-3 and Bax. Our findings are in accordance with previous research regarding the BITC mechanism in other cancers (Huang et al., 2012; Xiao et al., 2006). Moreover, addition of BIP-V5 could partially rescue the apoptosis induced by BITC treatments, suggesting BITC targets senescent IPF fibroblasts for Bax-dependent apoptosis.

AKT is a central component of the PI3K/AKT pro-survival pathway, and its aberrant activation contributes to apoptosis resistance. In several types of cancer cells, AKT has been shown to suppresses apoptosis induced by chemotherapeutics, oxidative and osmotic stress, and irradiation (Xu J. et al., 2023). In the present study, we confirmed that senescent IPF fibroblasts exhibited increased AKT activation compared with passage-matched control fibroblasts and early passage IPF fibroblasts. We further confirmed that downregulation of the AKT survival pathway may contribute to the senolytic activity of BITC. Although the precise mechanism by which BITC inhibits AKT activation remains unclear, our findings suggest that AKT could be a potential target for its senolytic effects. Previous studies have shown that HSP90, a molecular chaperone regulating AKT involved in cancer cell survival (Sun et al., 2024), plays an essential role in this pathway. However, our results indicate that BITC does not affect HSP90 expression in senescent fibroblasts (Supplementary Figure S5), suggesting that BITC may impact the AKT survival pathway via alternative mechanisms. Additionally, considering that autophagy is a general mechanism underlying age-related lung fibrosis and has been reported to be a target for BITC in lung cancer (Zhang et al., 2017), we cannot rule out the involvement of other regulatory pathways.

Preclinical animal models of lung fibrosis are usually used to preliminarily evaluate the therapeutic potential of drugs. Considering that IPF is reported to occur more frequently in males than females, male mice were selected for this study to establish the pulmonary fibrosis model (Iwai et al., 1994). Initially, we used young mice to investigate the effects of BITC *in vivo* and observed that BITC had no significant impact on BLM-induced lung fibrosis in young mice (Supplementary Figure S4). Interestingly, BITC treatment significantly attenuated lung fibrosis in aged mice, suggested that the antifibrotic effects of BITC are age-related. Fibroblasts in injured lung tissues of aged mice acquire a sustained senescent and apoptosis-resistant phenotype that impairs the resolution of fibrosis (Hecker et al., 2014). Our studies found that repeated treatment with BITC significantly attenuated the age-related persistent lung fibrosis and resulted in a reduction in senescent lung fibroblasts, suggesting that BITC had senolytic activity *in vivo*. To our knowledge, this is the first report revealing the anti-fibrotic and senolytic effects of BITC. Considering that the senescence of other cell types, such as alveolar epithelial cells, also contributes to the fibrogenesis of IPF, further studies should comprehensively evaluate the senolytic effects of BITC on these cells. This limitation highlights an area for further investigation in our study. Additionally, despite BITC’s safety being confirmed in several studies, some research has found that long-term supplementation with BITC may cause neutrophilia and hemoglobin reduction in mice (Kim et al., 2011). We did not observe significant adverse effects in mice in our study, but future safety assessments of BITC for the treatment of lung fibrosis are warranted.

## 5 Conclusion

In conclusion, our study for the first time, revealed that BITC have senolytic effects in senescent IPF fibroblasts and age-related lung fibrosis *in vivo*. BITC provokes senolysis through inducing senescent cell under Bax-dependent apoptosis and blocking AKT signal pathway. These findings may open a new avenue for the application of BITC to the therapy of IPF and other age-related organ fibrosis in the translation value of this natural compound.

## Data Availability

The original contributions presented in the study are included in the article/Supplementary Material, further inquiries can be directed to the corresponding authors.

## Abbreviations

α-SMA, α-smooth muscle actin; BAX, bcl2-associated X; BCL2, B-cell lymphoma 2; BLM, bleomycin; BITC, benzyl isothiocyanate; CDKN1A, cyclin-dependent kinase inhibitor 1A; CDKN2A, cyclin-dependent kinase inhibitor 2A; Cl. Casp. 3, cleaved caspase 3; COL1A1, collagen type I; COL3A1, collagen type III; IB, iberin; ITCs, isothiocyanates; IPF, idiopathic pulmonary fibrosis; PDL, population doubling level; SASP, senescence-associated secretory phenotype; SA-β-Gal, senescence-associated β-galactosidase; SCs, senescence cells; SFN, sulforaphane; Vim, vimentin; 4PI, 4-pentenyisothiocyanate.
