# Supplementary material for: Benzyl isothiocyanate provokes senolysis by targeting AKT in senescent IPF fibroblasts and reverses persistent pulmonary fibrosis in aged mice
Source: Front Pharmacol. 2025 May 2;16:1506518. doi: 10.3389/fphar.2025.1506518 (PMC12081420; doi:10.3389/fphar.2025.1506518)
Supplement: Supplementary file 1 [file DataSheet1.docx]

**Supplementary data and figures**

**Benzyl isothiocyanate provokes senolysis by targeting AKT in senescent IPF fibroblasts and reverses persistent pulmonary fibrosis in aged mice**

Rui Wang ^a #^, Fan Yang ^a #^, Ying Liu ^a^, Meiting Peng ^a^, Lu Yu ^a^, Qinhui Hou ^a^, Yuan Liu ^a *^ and Zhenshun Cheng ^a, b, c *^

^a^ Department of Respiratory and Critical Care Medicine, Zhongnan Hospital of Wuhan University, Wuhan, China

^b^ Wuhan Research Center for Infectious Diseases and Cancer, Chinese Academy of Medical Sciences, Wuhan, China.

^c^ Hubei Engineering Center for Infectious Disease Prevention, Control and Treatment, Wuhan, China.

^#^ These authors contributed equally to this work.

^*^ Corresponding author: Yuan Liu, E-mail: [liuyuanshi33@whu.edu.cn](mailto:liuyuanshi33@whu.edu.cn)

Zhenshun Cheng, E-mail: zhenshun_cheng@126.com

**Table S1.** Primer sequences used in experiments**.**

| Species | Primer | Forward (5’–3’) | Reverse (3’–5’) |
| --- | --- | --- | --- |
| Human | *β-actin* | TGCTATCCAGGCTGTGCTAT | AGTCCATCACGATGCCAGT |
|  | *CDKN1A* | CTCTCCCGAAAAGCAGTCCC | GCGAGAACGAGCCAACCTT |
|  | *CDKN2A* | GATCCAGGTGGGTAGAAGGTC | CCCCTGCAAACTTCGTCCT |
|  | *CCL2* | GCCTCTGCACTGAGATCTTC | AGCAGCCACCTTCATTCC |
|  | *IL6* | CGGCTACATCTTTGGAATCTTC | GCCCAGCTATGAACTCCTTC |
|  | *CXCL1* | CTGGCTTAGAACAAAGGGGCT | TAAAGGTAGCCCTTGTTTCCCC |
|  | *CXCL8* | TTTTGCCAAGGAGTGCTAAAGA | AACCCTCTGCACCCAGTTTTC |
|  | *FGF2* | AGAAGAGCGACCCTCACATCA | CGGTTAGCACACACTCCTTTG |
|  | *MMP2* | CTCATCGCAGATGCCTGGAA | TTCAGGTAATAGGCACCCTTGAAGA |
|  | *MMP11* | CCGCAACCGACAGAAGAGG | ATCGCTCCATACCTTTAGGGC |
|  | *MMP14* | CGAGGTGCCCTATGCCTAC | CTCGGCAGAGTCAAAGTGG |
| Mouse | *β-actin* | CATTGCTGACAGGATGCAGAAGG | TGCTGGAAGGTGGACAGTGAGG |
|  | *Cdkn1a* | CCTGGTGATGTCCGACCTG | CCATGAGCGCATCGCAATC |
|  | *Cdkn2a* | CGCAGGTTCTTGGTCACTGT | TGTTCACGAAAGCCAGAGCG |
|  | *Il6* | TCCTTAGCCACTCCTTCTGT | AGCCAGAGTCCTTCAGAGA |
|  | *Ccl2* | TAAAAACCTGGATCGGAACCAAA | GCATTAGCTTCAGATTTACGGGT |
|  | *Mmp2* | GACACCTGCACCACCTTA | AAGGTTGAAGGAAACGAGC |

**Table S2**. IPF Patients and control subject demographics.

| ID | Disease | Sex | Age | Smoking history | Pack-years in former smokers |
| --- | --- | --- | --- | --- | --- |
| IPF001 | IPF | 1-Male | 58 | Yes | 30 |
| IPF003 | IPF | 1-Male | 59 | No | / |
| IPF008 | IPF | 1-Male | 58 | Yes | 45 |
| Con001 | Normal | 2-Female | 56 | No | / |
| Con002 | Normal | 1-Male | 69 | Yes | 40 |
| Con005 | Normal | 1-Male | 60 | Yes | 30 |

**Supplemental Figures**

**Figure S1**

**
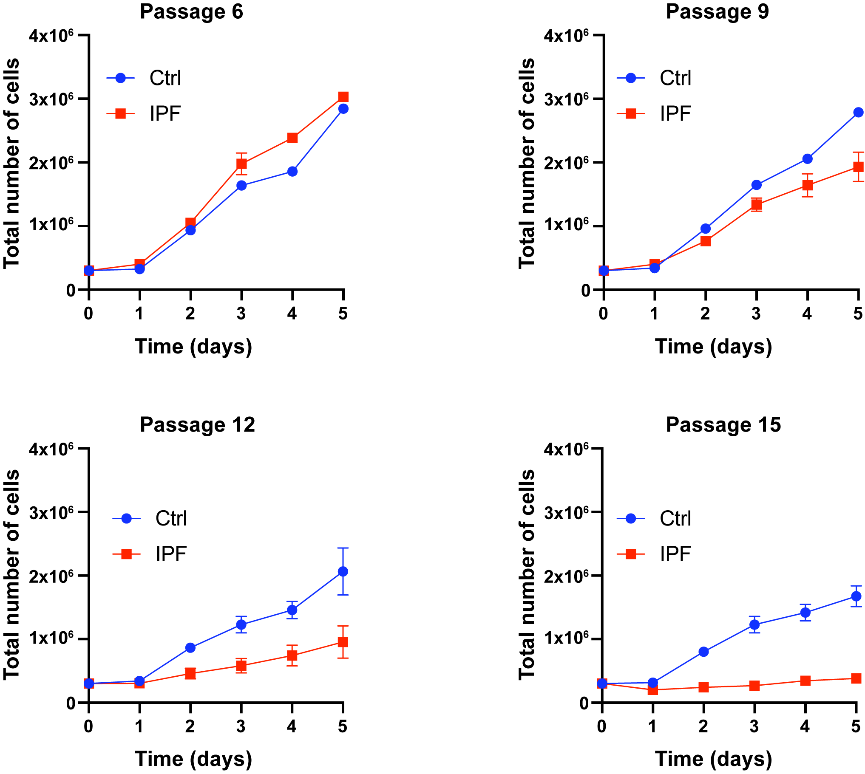
**

**Figure S1.** Growth rates of Control and IPF lung fibroblasts in regular culture conditions. Cells were seeded in triplicates at the indicated passage levels, the cultures were harvested on the indicated days and the amounts of cells per dish obtained.

**Figure S2**


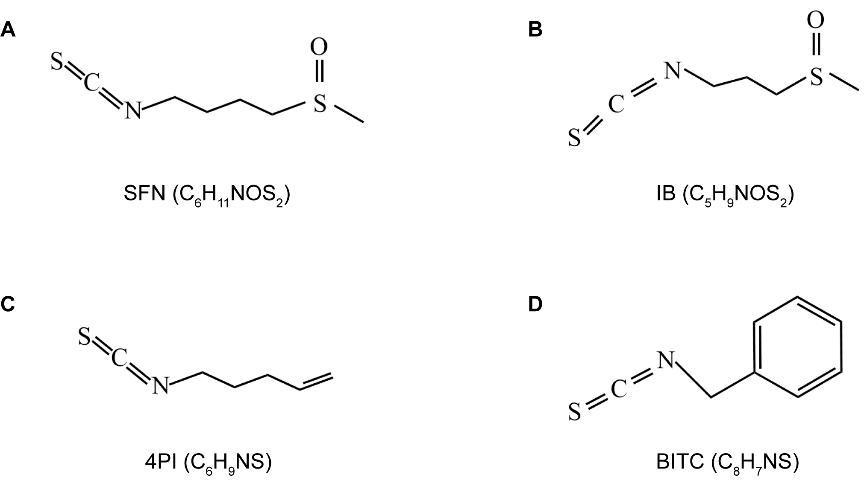


**Figure S2.** Chemical structures of the compounds used for screening experiments. (A) Sulforaphane (SFN). (B) Iberin (IB). (C) 4-pentenyisothiocyanate (4PI). (D) Benzyl isothiocyanate (BITC).

**Figure S3**


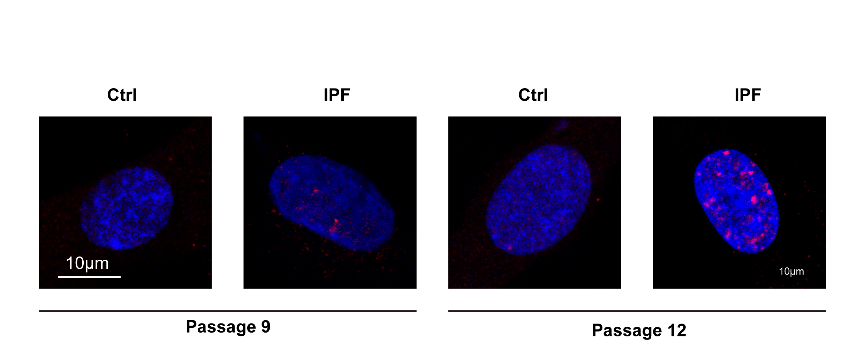


Figure S3. Representative images of 53BP1 foci in control (Ctrl) and IPF lung fibroblasts at passage 9 level and passage 12 level.

Figure S4


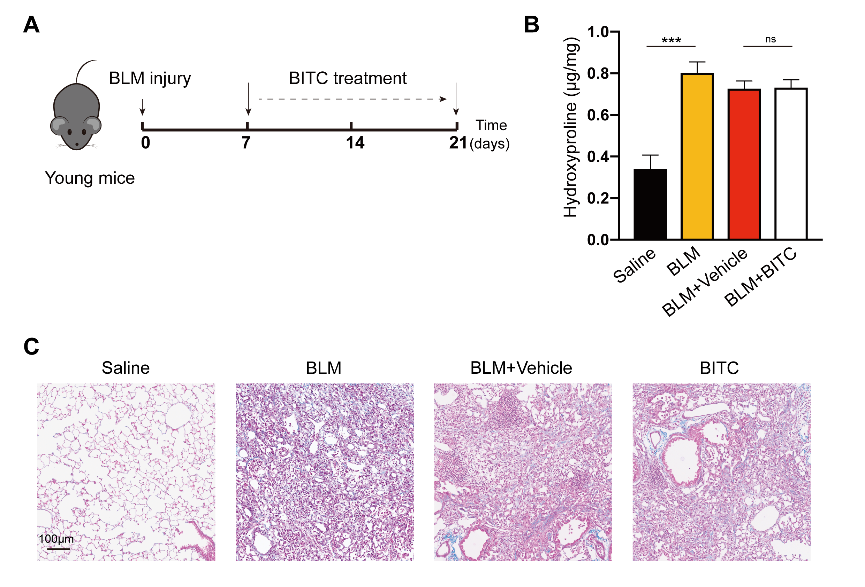


Figure S4. BITC had no effect on BLM-induced lung fibrosis in young mice. (A) Modeling diagram. (B) The hydroxyproline content in the lung tissues. (C) Representative images of Masson staining. scale bar=100μm. Error bars indicate mean ± SEM. n = 6 per group. Significant differences were assessed by two-way ANOVA. ***P ＜ 0.001.

ns = no significant.

Figure S5


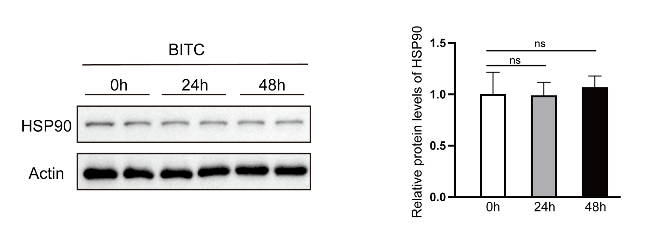


Figure S5. BITC had no effect on the protein levels of HSP90 in senescent IPF lung fibroblasts. Senescent IPF fibroblasts were treated with BITC for 24 h and 48 h, the expression of HSP90 protein levels were detected by western blotting. Error bars indicate mean ± SEM. Significant differences were assessed by one-way ANOVA. ns = no significant.

**Raw image for western blots**

**
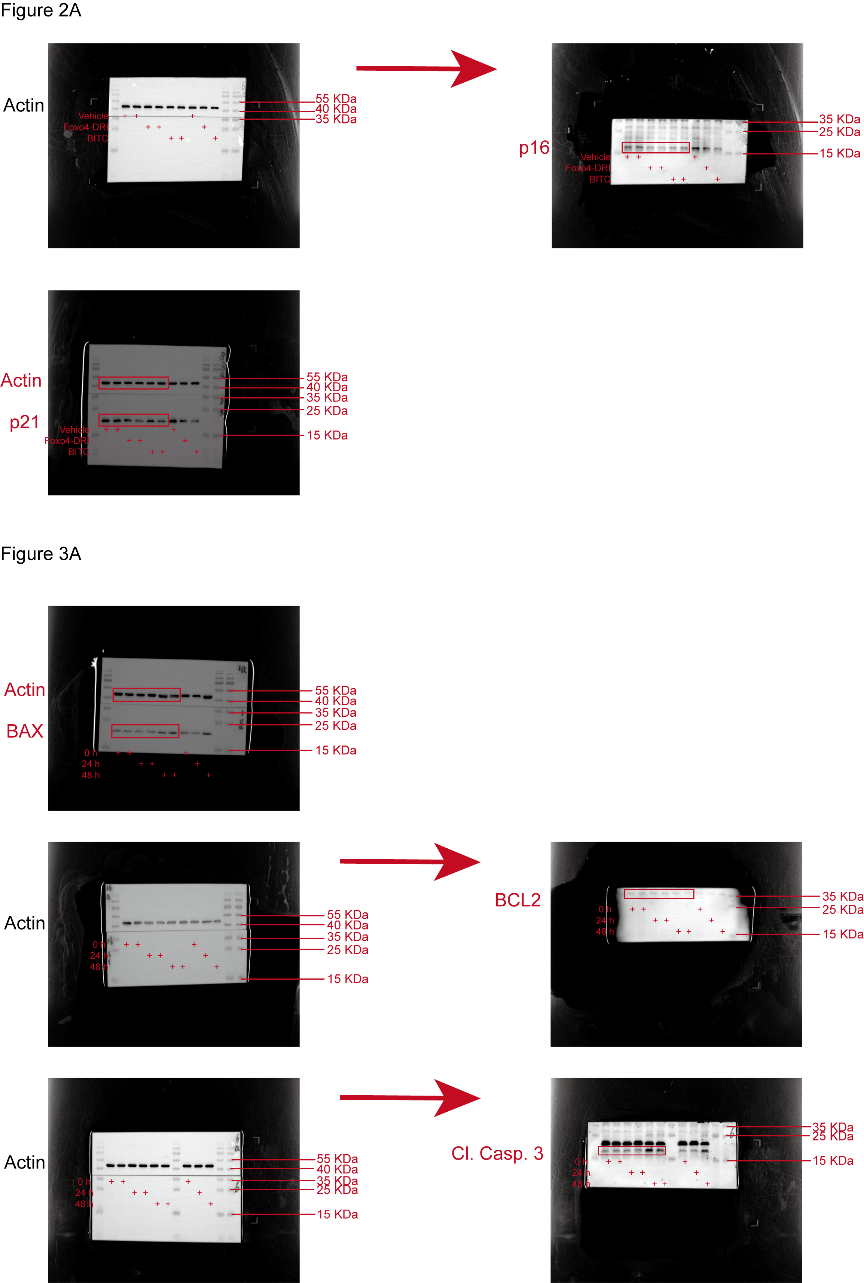
**

**
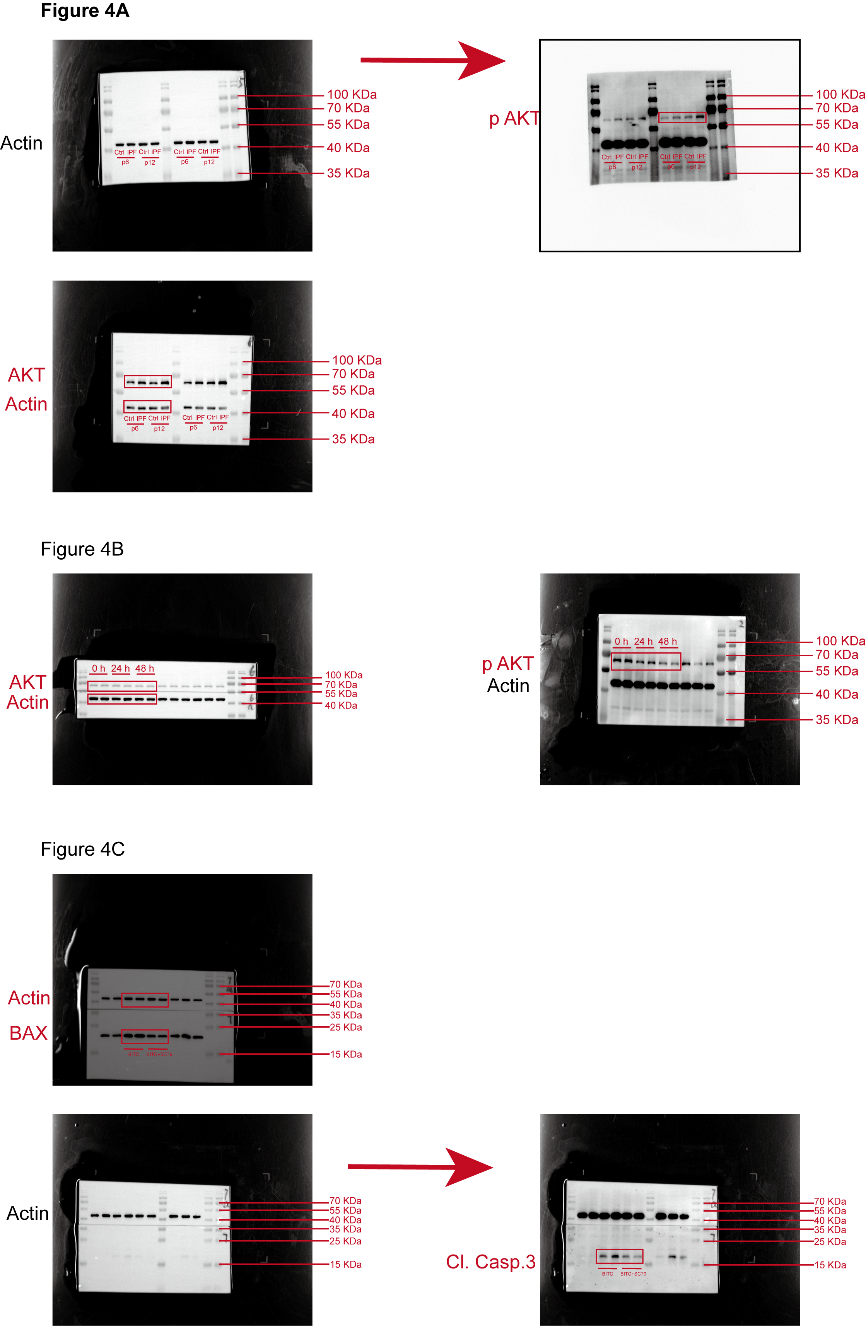
**

**
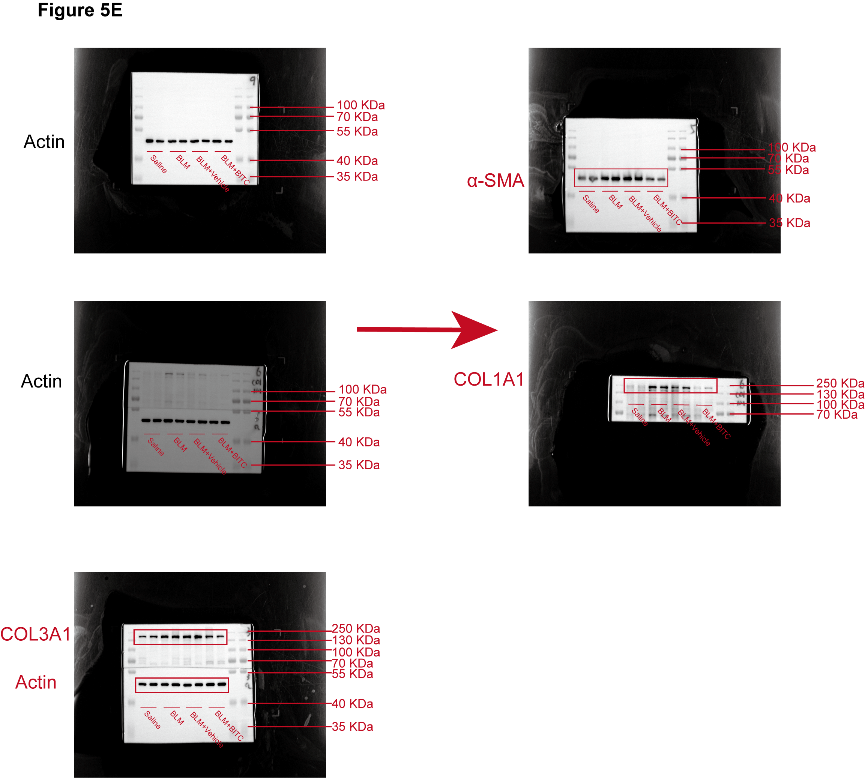
**

**
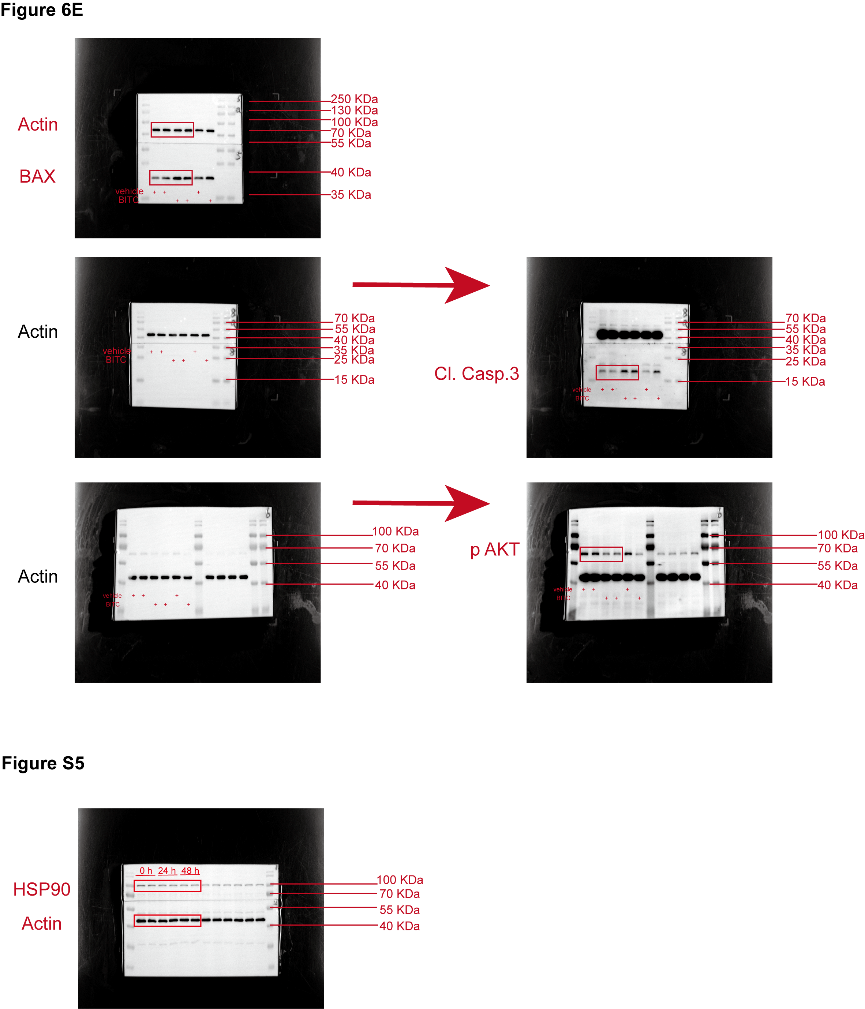
**
